# Diverse mechanisms shape the evolution of virulence factors in the potato late blight pathogen *Phytophthora infestans* sampled from China

**DOI:** 10.1038/srep26182

**Published:** 2016-05-19

**Authors:** E-Jiao Wu, Li-Na Yang, Wen Zhu, Xiao-Mei Chen, Li-Ping Shang, Jiasui Zhan

**Affiliations:** 1 Fujian Key Laboratory of Plant Virology, Institute of Plant Virology, Fujian Agriculture and Forestry University, Fuzhou, Fujian, P. R. China; 2 Key Lab for Biopesticide and Chemical Biology, Ministry of Education, Fujian Agriculture and Forestry University, Fuzhou, Fujian, P. R. China

## Abstract

Evolution of virulence in plant pathogens is still poorly understood but the knowledge is important for the effective use of plant resistance and sustainable disease management. Spatial population dynamics of virulence, race and SSR markers in 140 genotypes sampled from seven geographic locations in China were compared to infer the mechanisms driving the evolution of virulence in *Phytophthora infestans* (*P. infestans*). All virulence types and a full spectrum of race complexity, ranging from the race able to infect the universally susceptible cultivar only to all differentials, were detected. Eight and two virulence factors were under diversifying and constraining selection respectively while no natural selection was detected in one of the virulence types. Further analyses revealed excesses in simple and complex races but deficiency in intermediate race and negative associations of annual mean temperature at the site from which pathogen isolates were collected with frequency of virulence to differentials and race complexity in the pathogen populations. These results suggest that host selection may interact with other factors such as climatic conditions in determining the evolutionary trajectory of virulence and race structure in *P. infestans* and global warming may slow down the emergence of new virulence in the pathogen.

How virulence evolves in nature is still unclear and has been a central theme in evolutionary studies of host-pathogen interactions. Here, virulence is defined as the ability of a pathogen strain to circumvent the defense response of a plant carrying a corresponding resistance (R) gene^1^. In agricultural systems, this knowledge is important in race-specific R gene breeding and designing effective disease management strategies. Many plant pathogens interact with their hosts according to the widely accepted gene-for-gene model, which assumes that plants only express disease resistance when a R gene product in the host can recognize, directly or indirectly, a specific effector gene product in the pathogen^2^. This theory assumes that virulence is required by pathogens to exploit hosts and ensure successful invasion, growth, reproduction and transmission^3^ when the corresponding R gene is present in the host population. However, virulence may also pose a fitness penalty to survival and reproduction of pathogens. For instance, compared to avirulent pathotypes, pathogens carrying virulence genes may have lower aggressiveness and competitiveness when the hosts do not have a corresponding R gene and adapt less well to other biotic and abiotic stresses^4^. The fitness cost model predicts a quick increase in the frequency of virulence when a new R gene is widely adopted and gradual decline of frequency in virulence when the corresponding R gene is withdrawn from commercial use.

Though there are many recorded examples of fitness costs in the plant pathology literature^5^,^6^, such penalties for carrying unnecessary virulence are not always detected^4^. However, a lack of demonstrated fitness costs in these cases does not necessarily conflict with predictions for evolution of virulence based on the gene-for-gene model. Rather, it suggests that other biological, ecological and evolutionary processes may interact with host selection in determining the evolutionary trajectory of virulence in nature. In an ecological context, the survival, reproduction and transmission of plant pathogens is determined not only by hosts but also by their interaction with other abiotic and biotic factors such as temperature, humidity and the genetic composition and abundance of other host and non-host species^7^. In an evolutionary context, the efficiency of natural selection can be influenced by effective population sizes, spatial structure, mating system and pathogen host range^8^. Selection against unnecessary virulence could also be confounded by random genetic drift due to small effective population sizes associated with population bottlenecks or founder events during the pathogen life cycle. In addition, linkage disequilibrium generated by non-random mating and compensatory mutations may also be important in shaping the evolutionary trajectory of virulence^6^. Under linkage disequilibrium, unnecessary virulence genes could be maintained in pathogen populations by hitchhiking with alleles at other loci that determine higher parasite fitness, though the level of this indirect selection might be reduced in some virulence genes experiencing frequent sequence rearrangements^9^. Linkage disequilibrium could be particularly important in the plant pathogens with limited sexual reproduction.

Here, we used potato-late blight system to test the hypothesis that the evolution of virulence factors in pathogens is driven by multiple mechanisms. Potato (*Solanum tuberosum L*.) is the third-ranked food crop in total production globally^10^. Late blight caused by *Phytophthora infestans* (Mont) de Bary is the most destructive disease of potato, responsible for the Irish potato famine in the 1840s and still being the most devastating disease worldwide^11^, particularly in areas experiencing moderate temperature and high humidity. The pathogen can affect all parts of potato crops including leaves, stems and tubers. Under favorable climatic conditions, an entire potato crop can be destroyed within a few days. The annual economic loss caused by potato late blight is ~$6.7 billion in the world^12^.

Host resistance has been thought to be a cost-effective and environmentally safe approach to manage plant diseases including potato late blight^13^,^14^. Novel sources of race-specific resistance to many pathogens have proved to be very effective when they are first introduced into commercial cultivars. However, this race-specific resistance often becomes ineffective after two to three growing seasons due to evolution of pathogen populations from avirulence to virulence^15^ and the formation of new physiological races, here defined as a subgroup of pathogens within a species that infect plant varieties carrying a given set of R genes. Indeed, it has been documented that apparent increases in the frequency and severity of late blight epidemics and many other diseases in recent years^16^ have resulted from the emergence of new physiological races able to overcome resistances widely utilized in crop production^17^. In potato at present, 11 race-specific resistance genes from *S. demissum* have been identified^18^, as well as some broad-spectrum resistance^17^ from other *Solanum* species. However, large numbers of these race-specific resistances are not effective due to the presence of corresponding virulence in the pathogen.

Virulence factors and race structure have important effects on the effectiveness and durability of potato and other crops with race-specific resistance. As a consequence, substantial efforts have been made to survey the temporal and spatial distribution of virulence factors and race structure of pathogens^19^. Some of these surveys cover large geographic areas^20^ and have continued for a long time^21^. However, the majority of these surveys have been restricted to simple descriptions of frequency distribution in virulence factors and race structure (but see^22^). According to our knowledge, empirical assessments of the ecological and evolutionary mechanisms driving the population dynamics of plant pathogens through the comparative analysis of spatial distribution in virulence factors, physiological races and neutral markers across multiple locations have not been done yet, specially for *P. infestans*. Information derived from this type of study is needed to increase the durability of R genes and minimize the risk of breakdown in host resistance. Therefore, the objectives of this study are to compare spatial distributions of virulence factors, race characters and neutral variation in *P. infestans* using a combination of a molecular assay with selectively neutral SSR markers and biological assays on differential potato cultivars with intentions to 1) infer the effect of natural selection on the evolution of virulence factors and race structure in plant pathogens; and 2) evaluate the dependence of virulence and race frequencies in plant pathogens on local temperature condition.

## Results

### Population genetic structure in SSR marker loci

**Figure 1:**
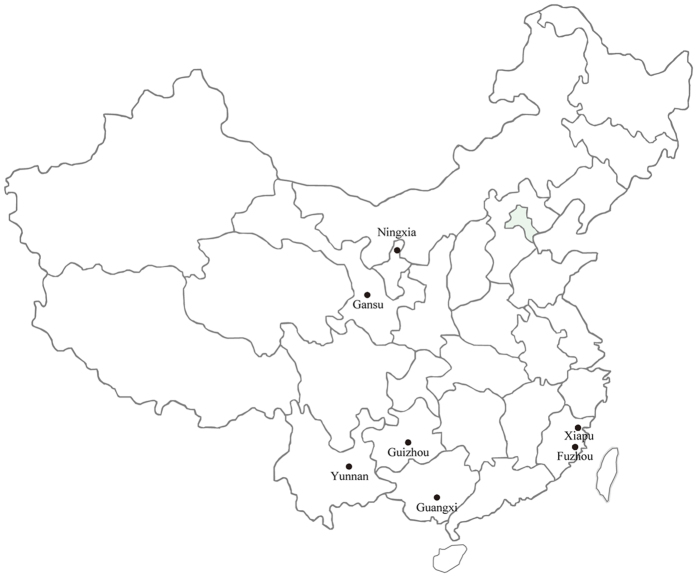
Map showing the geographic locations of the seven *Phytophthora infestans* populations included in this study. The map was generated by ArcGIS 9.3 software (Environmental Systems Research institute, Redlands, CA, USA) (http://www.arcgis.com).

The expected heterozygosity across the eight SSR marker loci in the seven geographic populations of *P. infestans* in China (Fig. 1) ranged from 0.396 to 0.498 with a grand expected heterozygosity of 0.457 when the 140 isolates from the seven populations were combined. In contrast, the observed heterozygosity across the eight SSR marker loci ranged from 0.724 to 0.863 (Table 1) with a grand observed heterozygosity of 0.748 when the 140 isolates from the seven populations were combined. In all populations, observed heterozygosity was higher than expected. The *P. infestans* population sampled from Xiapu displayed the highest level of both expected and observed heterozygosity. All field populations showed significantly gametic disequilibrium among SSR markers (Table 1).

### Population genetic structure in virulence

**Figure 2:**
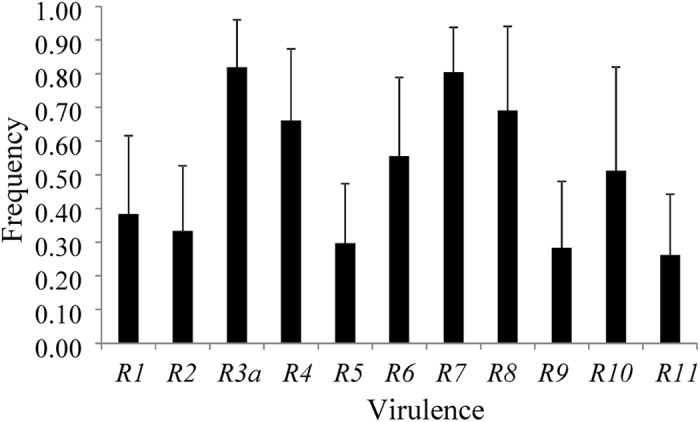
Virulence frequencies and their 95% confidence intervals in the 140 *Phytophthora infestans* sampled from seven geographic locations in China.

Frequencies of all virulence differed significantly among the seven populations (Table 2). Among the 11 types of virulence, avr*3a* and *avr7* were at the highest frequency, ranging from 0.60 to 1.00 and 0.65 to 1.00 with an average of 0.82 and 0.81, respectively (Table 2, Fig. 2). On the other hand, avr*5, avr9* and *avr11* were at the lowest frequency, ranging from 0.00 to 0.45, 0.05 to 0.55 and 0.00 to 0.50 with an average of 0.30, 0.29 and 0.26, respectively. In this survey, *avr10* displayed the highest fluctuation in frequency among populations, ranging from 0.15 in Fuzhou to 0.90 in Ningxia, with an average of 0.51. All isolates from Ningxia carried *avr3a* and *avr7* and therefore were able to overcome the differential cultivars carrying *R3* and *R7.* No isolates from Fuzhou have *avr5* and *avr11* to overcome differential cultivars carrying *R5* and *R11.*

Phenotypic diversity in the population pooled from the seven locations ranged from 0.293 for *avr3a* to 0.500 for *avr10* (Table 1). At the individual population level, the highest average phenotypic diversity (0.421) across the 11 types of virulence was observed in Yunnan while the lowest (0.280) was observed in Fuzhou. Population differentiation (*G*_*ST*_) for the 11 types of virulence ranged from 0.094 for *avr7* to 0.320 for *avr10* (Table 3). All *G*_*ST*_ values for the virulence except *avr3a, avr5* and *avr7* were significantly higher than the population differentiation (*F*_*ST*_) in SSR marker loci. *G*_*ST*_ values for *avr3a* and *avr5* did not differ significantly to *F*_*ST*_ and *G*_*ST*_ values for *avr7* were significantly lower than *F*_*ST*._

### Population genetic structure in physiological race

**Figure 3:**
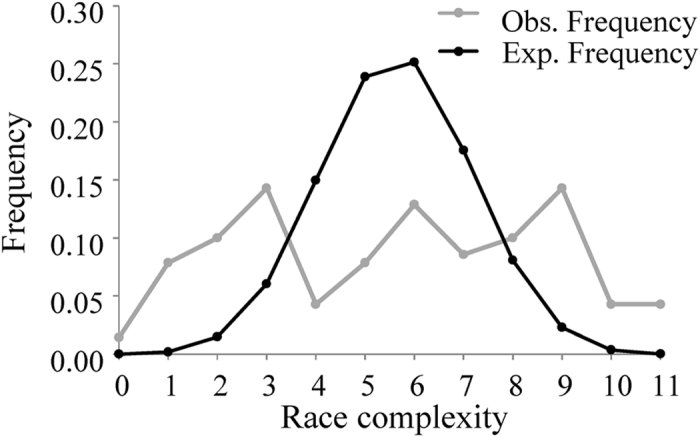
Observed and expected frequency of race complexity in the 140 *Phytophthora infestans* isolates from seven geographic locations in China.

16–19 race types were detected in each population with a total of 89 physiological races detected in the pooled population. Standardized Shannon index of physiological race diversity in the seven populations ranged from 0.91 to 0.98 with a pooled Shannon index of 0.99 when the 140 isolates from the seven populations were combined (Table 4). Among the 89 physiological races detected, 62 (70%) were observed only once and 67 (75%) were observed in one location (Fig. 3) only. The two most common physiological races were observed six times (Fig. 3). One of them was able to induce disease on all 11 differential cultivars and was also the most widespread race observed in five of the seven locations assayed).

**Figure 4:**
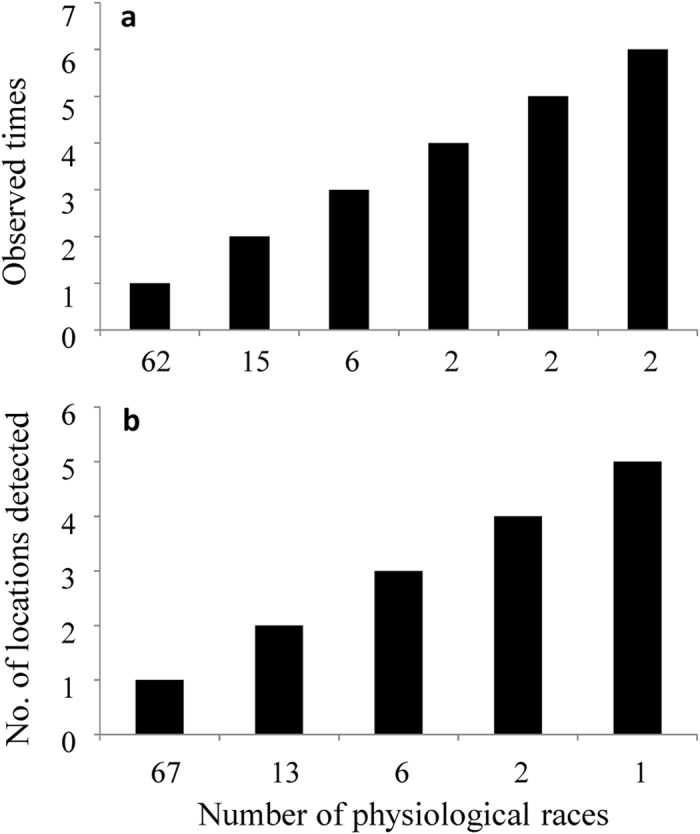
Frequency and spatial distribution of physiological races in the 140 *Phytophthora infestans* isolates collected from seven geographic locations in China: (**a**) times of physiological races observed; and (**b**) locations of physiological races observed.

**Figure 5:**
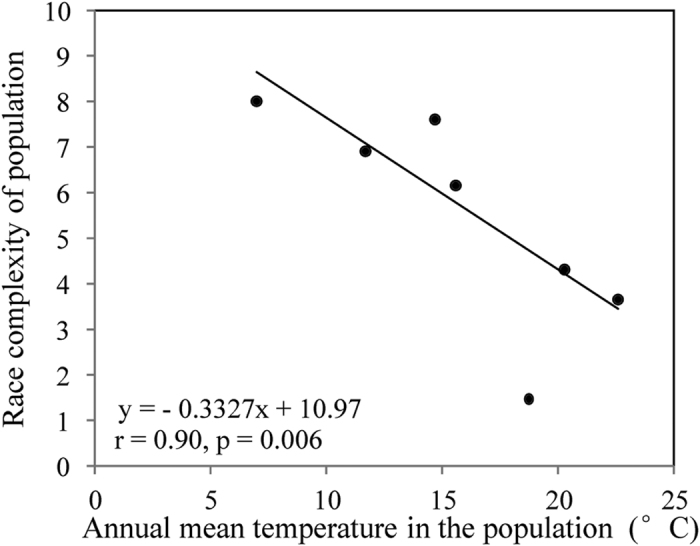
Correlation of the annual mean temperature in the sites the *Phytophthora infestans* populations were collected with the mean race complexity of the pathogen populations tested on 11 differentials each with one major R derived from *Solanum demissum*.

A full spectrum of pathogen race complexity, ranging from 0 to 11 virulence factors per isolate, was detected. Average race complexity in local populations differed significant among populations collected from different locations ranging from 2.28 to 8.00 with a grand mean of 5.77 when all isolates were pooled into a single population (Table 4). The highest race complexity was found in *P. infestans* sampled from Ningxia while the lowest race complexity was found in the Fuzhou population. The observed frequencies of simple (≤3.0 virulence types) and complex (≥8.0 virulence types) races were higher than expected while the observed frequency of races with intermediate complexity (4–7 virulence types) was lower than expected (Fig. 4). In all but one case, genetic variation in SSR marker loci did not correlate with phenotypic variation in virulence factors (Table 5). Annual mean temperatures at the collection sites were negatively correlated with the frequencies of virulence (Table 5) and race complexity (Fig. 5, r_5_ = −0.90, p = 0.006) in the populations.

## Discussion

Understanding the spatial dynamics of virulence factors and pathogen physiological races as well as mechanisms underlying their emergence and evolution is important for the effective use of major gene resistance in sustainable disease management. In this study, we analysed population genetic dynamics of 140 *P. infestans* isolates to investigate the evolutionary mechanisms of virulence and race characteristics and their dependence on local temperature. These isolates were sampled from seven locations across the main potato production areas in China. Molecular assays of variation were done with eight SSR markers and phenotypic assays of virulence were done using a standard set of 11 potato differential cultivars derived from *S. demissum*^23^. All virulence types and the full spectrum of race complexity were detected and ~5% of the isolates were able to induce disease on all 11 resistance genes (Figs 2 and 3). These results indicate a high frequency of virulence factors and complicated race structure in the *P. infestans* populations, consistent with previous reports on the pathogen^19^.

In gene-for-gene interactions, host selection has been thought to be the fundamental force driving the evolution of virulence factors and race structure. Due to the fitness cost of unnecessary virulence genes, the frequency of virulence factors and complex races in the absence of corresponding resistance genes or resistance gene combinations such as R gene pyramids, cultivar mixtures or breeding materials from composite crosses is expected to decline quickly^2^,^24^. In China, late blight has been controlled primarily by cultivars with single resistance genes or partial resistance, complemented by the application of fungicides^25^,^26^ and most of the 11 resistance genes derived from *S. demissum* have never been used on a commercial scale. Our result suggests that except host selection, other mechanisms such as environmental factors and pathogen life-history traits may also function together with host selection in shaping the emergence and evolution of virulence and race characters in *P. infestans* as reported in other plant and animal pathogens^27^. The Interaction among these various evolutionary mechanisms may also explain the lack of correlation between genetic variation in SSR marker loci and virulence in this study (Table 5) and an inability to detect fitness costs of unnecessary virulence in many other pathogens^4^.

Indeed, when population differentiation in SSR marker loci and virulence was compared, we found three evolutionary patterns in virulence factors (Table 1). The majority of virulence factors had a higher population differentiation than SSR marker loci, suggesting these virulence factors are under diversifying selection according to environmental conditions^28^ including the use of R genes in each geographic location. Virulence to *R5* (*avr5*) behaved as a selectively neutral marker as indicated by its similar level of population differentiation with SSR marker loci while *avr7* had a lower population differentiation than SSR markers, suggesting this virulence factor is under constraining selection^28^, i.e. different geographic locations either all select for or select against the virulence factor. Genetic variation in *avr3a* was low both in this and previous studies^29^. This is unexpected for a gene involved in host-pathogen antagonistic interactions and may represent the consequences of a selective sweep over its evolutionary history^30^. Different patterns of selection were also found in avirulence gene of the fungal pathogen *Melampsora lini* by sequence analysis^31^.

Though we found *G*_*ST*_ for the *avr3a* was lower than *F*_*ST*_ for SSR markers, the difference is not significant. The estimate of population differentiation using *F*-statistics tends to be biased upwards when within-population diversity is low and biased downwards when within-population diversity is high^32^. When we corrected for these biases with a new analytic method^33^, we found that *G*_*ST*_ for *avr3a* was significantly lower than that for SSR marker loci, consistent with the hypothesis of a selective sweep in this virulence factor, but patterns on the difference between *G*_*ST*_ and *F*_*ST*_ did not change for other virulence factors (data not shown).

In this analysis, we treated each virulence factor as a bi-allele locus. However, recent analyses indicated that some of the Black differentials^34^ may harbor multiple R genes. But we do not believe that the current development in the resistant nature of the differentials would largely affect our conclusions on the evolutionary patterns of the virulence factors. For example, *R3* differential was shown to contain two closely linked R genes (*R3a* and *R3b*^35^). Due to clonal reproduction of *P. infestans*, these two genes are highly possible to inherit like a single locus. It was also reported that the *R9* differential may harbor *R1* and other R genes^36^. If these results are confirmed, isolates able to induce late blight in the *R9* differential are expected to also induce the disease in the *R1* differential because the latter was shown to harbour only R1 gene^37^. Interestingly, in the current study many isolates which produced the late bight disease on the *R9* differential did not produce the disease on *R1* differential.

Abiotic factors such as air temperature may represent another major driver of the evolution of virulence factors and race complexity in *P. infestans*. Temperature is one of the most important environmental parameters which can have crucial impacts on nearly all aspects of biological processes^38^. In host-pathogen interactions, temperature can have not only a critical influence on the occurrence and severity of disease epidemics in the short-term but also on the longer-term evolutionary trajectory of pathogens^39^. The hypothesis of temperature mediated dynamics of virulence factors and race structure in *P. infestans* is supported by significant associations of virulence frequency and race complexity in pathogen populations with the mean annual temperature in the sites where pathogen populations were sampled (Table 5, Fig. 3). Overall, *P. infestans* populations originating from cooler places had higher virulence frequency and race complexity than those from warmer places in China though many of these cooler regions (e.g. Ningxia and Gansu) are not conducible to repeated outbreaks of the late blight disease due to drier weather, therefore supporting smaller pathogen populations and providing fewer opportunities for virulent races to evolve. Therefore, our observed pattern of virulence evolution is unlikely to be due to small pathogen population sizes under warm conditions. Rather, it suggests that virulence factor may have reduced fitness under high temperatures though the genetic basis of this fitness cost is not clear and further tests in other pathogens are required to generalize the fitness cost hypothesis. Plant pathogen avirulence genes encode diverse proteins with a range of demonstrated or postulated physiological functions^40^,^41^ involved in life history traits which may have a gall-inducing effect on adaptation to abiotic and biotic environments including temperature. In *Agrobacterium tumefaciens*, a bacterial pathogen of plants, it has been demonstrated that key virulence factors are often repressed in response to elevated temperature^42^,^43^.

Linkage disequilibrium generated by asexual reproduction may be another major force determining the evolution of virulence factors and race complexity in *P. infestans.* Linkage disequilibrium could be particularly important in Chinese populations of *P. infestans*, in which the contribution of sexual reproduction to the population genetic structure of the pathogen is limited though self-fertile genotypes have dominated the populations^44^. Indeed, when we conducted gametic disequilibrium and genetic variation analyses, we found significant heterozygosity excess, an indication of asexual reproduction, and non-random association of SSR loci in all populations (Table 1). Under asexual reproduction, the frequencies of unnecessary virulence once dominant in pathogen populations could be ‘fossilized’ when corresponding resistances are withdrawn from commercial use due to lack of mechanisms to remove them or hitchhiking with alleles at other loci that have a higher fitness. This would lead to a gradual increase of race complexity^4^. Indeed, it has been reported that pathogens with the greatest race complexity are usually found in clonal populations of pathogens faced with long-standing race-specific resistances^45^ while those with the least race complexity are found in populations recently challenged by new resistances^46^. The combination of fossilization for high race complexity and biotic selection from continuous introduction of simple resistance for simple race complexity may contribute to the observed patterns of excesses in simple and complex races and deficiency in intermediate race (Fig. 3) in *p. infestans* and other pathogens^47^.

Our results provide clear evidence that multiple forces determine the evolution of virulence and race structure in *P. infestans*. This finding has important implications to breeders, extension specialists, plant pathologists and farmers in designing management strategies for controlling late blight and other major plant diseases with a similar pattern of virulence evolution. Resistance gene pyramids have been thought to be advantageous over traditional approaches of deploying single resistance genes for sustainable disease management^48^. The finding that pathogens can develop complex race structures in agricultural systems reinforces the likelihood that this strategy of managing resistance genes may not be sustainable. More dynamic disease management strategies such as engineering series of isogenic lines differing only in resistance genes and using them alternately^49^ or using a combination of major and partial resistance genes may be necessary.

As a result of anthropogenic activities, average temperatures are expected to increase several degrees in coming decades^50^. It is concerning that such global trend in air temperature may intensify disease occurrence in agriculture^51^. The negative associations of virulence frequency and race complexity with mean annual temperature in the current study raises the possibility that increases in air temperature during global warming may slow down the emergence of new virulence in *P. infestans* and other pathogens. If this eventuated it would increase the lifespan of resistant cultivars and reduce the costs of controlling late blight and other important pathogens in agriculture. This issue is worthy of further investigation.

## Materials and Methods

### Phytophthora infestans collections

Seven *Phytophthora infestans* populations collected along a climatic gradient representing various potato cropping zones in China during the 2010 and 2011 growing seasons^52^ were included in the study. Cultivars used in these fields include Hezuo No. 88, Kexin No. 1, Longshu No. 3 and Zhongshu No. 3. These cultivars do not have the known resistance genes from *S. demissum.* For all collections, infected leaves were sampled at random from plants separated by 1–2 meters and only one infected leaf was sampled from each plant. After collection, the leaf samples were immediately placed in separate sandwich bags to prevent cross-infection, and transferred within 24 hours to the laboratory for pathogen isolation. One single-spore strain was isolated from each infected leaf. Genotypes of these isolates were previously determined by SSR assay of nuclear genomes^53^,^54^, restriction enzyme-PCR amplification of mitochondrial haplotypes^55^, mating type^44^ and partial sequence analysis of three genes (β-tubulin, Cox1 and Avr3a)^56^ and a total of 140 distinct genotypes, with 20 in each of the seven field populations were selected for virulence factors and race structure. The details for pathogen isolation, molecular characterizations of these populations can be found in our previous publications^44^,^52^,^57^.

### Virulence assay

Virulence factors and physiological race on 11 differential potato cultivars each expressing one of the 11 known R genes from *S. demissum* and one universal susceptible cultivar were tested using a detached leaflet assay on 2% water-agar plates. The differentials were produced by backcross introgression of the 11 major R gene from the wild hexaploid species *S. demissum* to potato^34^ and were kindly provided by Prof. Qinghe Chen in the Institute of Plant Protection, Fujian Academy of Agricultural Science in Fuzhou, Fijian, P. R. China). Before virulence testing, the isolates were first inoculated onto leaflets from the universal susceptible cultivar to restore the pathogenicity of *P. infestans* which may have been lost due to long-term storage on agar. After inoculation at 18 °C for 10 days with 16h light supplementation daily, newly formed sporangia were washed from the leaflets using 5 mL chilled sterile distilled water and diluted to 4 × 10^5^ sporangia/mL with a Fuchs-Rosenthal haemocytometer. Droplets (5 μL) of sporangial suspensions were applied onto the abaxial side of detached leaves collected from the differential cultivars and the universal susceptible cultivar. Pathogen infection types were recorded after seven days inoculation at 18 °C in an incubator supplemented with 16 h light daily as described earlier. For this experiment, four replicates were done for each pair of isolate-differential interaction and an isolate was considered to carry a virulence factor towards a specific R gene if the inoculation spots on the detached leaf of the differential cultivar showed visual necrosis.

### Data analysis

Genetic variation and population differentiation in SSR markers for the seven pathogen populations were taken from a previous publication^52^. In these analyses, genetic variation in SSR marker loci was quantified by observed and expected heterozygosity. Population differentiation of the SSR marker loci was estimated by fixation index (*F*_*ST*_, using POPGENE 1.32 (http://www.ualberta.ca/~fyeh/popgene_download.html). The frequency of virulence was tabulated according to individual pathogen populations as well as the pooled population by combining all 140 *P. infestans* isolates from different locations and compared by a contingency χ2 test^58^. Phenotypic variation in virulence was quantified by Nei’s diversity and partitioned into within- and among-population components to estimate population differentiation^59^, also using POPGENE^60^. The evolutionary history of virulence factors was inferred by comparing their population differentiation index with *F*_*ST*_ in SSR marker loci. Diversifying selection for local environments is expected to increase genetic differentiation among geographic populations for virulence factors, leading to a significantly higher *G*_*ST*_ in the virulence characters than *F*_*ST*_ in SSR marker loci. Constraining selection is expected to decrease genetic differentiation among geographic populations for virulence factors, causing a significantly lower *G*_*ST*_ in the virulence characters than *F*_*ST*_ in SSR marker loci. If *G*_*ST*_ in the virulence factors and FST in SSR marker loci is not significantly different, the hypothesis of neutral evolution for the studied virulence factors is retained. The percentile of *G*_*ST*_ was generated by 1000 bootstraps of the original data with Resampling 6.20 as described previously^60^ and used to evaluate the statistical difference between the population differentiation in SSR marker loci and virulence.

Isolates were classified into races by integrating the virulence present at each locus. An isolate was considered to carry a virulence factor towards a particular R gene (differential cultivar) if it induced late blight symptoms. The expected frequency of physiological races was calculated using the frequencies of virulence detected in the current study. Thus if the frequency of virulence at locus j is *P*_*j*_, then the expected frequency of a physiological race possessing virulence at the *j*th locus across all loci will be 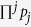. For example, the expected frequency of a physiological race containing virulence factors at all 11 loci will be the product of the frequency of virulence factors at every locus. Race diversity was calculated as a standardized Shannon index^61^.

Race complexity of an isolate was determined according to the number of differential cultivars on which the isolate could induce late blight disease. A complexity index of “11” indicates that the isolate could infect all 11 differential cultivars while a complexity index of “0” indicates that the isolate was able to induce late blight disease on the universally susceptible cultivar only. All isolates carrying the same number of virulence factors were treated as having the same race complexity regardless of the combination of differential cultivars they showed virulence against. With 11 differential cultivars, there are 12 potential types (0–11) of race complexity. These classes of race complexity were arbitrarily divided into three groups, each containing an equal number of four types. Isolates with a complexity index of ≤3, 4–7 and ≥8 were grouped into “simple”, “medium” and “complex” races, respectively. Fisher’s least significant difference (LSD) was used to compare race complexity among the pathogen populations collected from different locations. Meteorological data for each collection site were downloaded from World Climate (http://www.worldclimate.com/) to determine the associations of annual mean temperature and total rainfall with the evolution of virulence factors and race structure. Annual temperature s were estimated based on the mean temperature for each month^28^. Pearson correlation^62^ was used to evaluate the associations of annual mean temperature at collection sites with virulence frequency and race complexity and between genetic variation in SSR marker loci and phenotypic variation in virulence.

## Additional Information

**How to cite this article**: Wu, E. J. *et al*. Diverse mechanisms shape the evolution of virulence factors in the potato late blight pathogen *Phytophthora infestans* sampled from China. *Sci. Rep.* **6**, 26182; doi: 10.1038/srep26182 (2016).

## Figures and Tables

**Table 1:** Genetic variation of SSR marker loci (heterozygosity) and virulence factors (Nei’s diversity) and Hardy-Weinberg equilibrium (HWE) tests for non-random association (p value in parenthesis) in the seven *P. infestans* populations each composed of 20 genetically distinct isolates sampled from China.

Population	Sample size	SSR			Virulence factor											
		Obs.	Exp.	HWE	*avr1*	*avr2*	*avr3a*	*avr4*	*avr5*	*avr6*	*avr7*	*avr8*	*avr9*	*avr10*	*avr11*	Mean
Fuzhou	20	0.724	0.450	0.000	0.255	0.095	0.480	0.480	0.000	0.480	0.455	0.420	0.095	0.320	0.000	0.280
Gansu	20	0.725	0.483	0.000	0.480	0.480	0.095	0.375	0.495	0.480	0.180	0.255	0.420	0.420	0.480	0.378
Guangxi	20	0.735	0.446	0.000	0.095	0.255	0.375	0.480	0.255	0.375	0.320	0.495	0.375	0.375	0.255	0.332
Guizhou	20	0.734	0.411	0.000	0.495	0.480	0.180	0.255	0.480	0.420	0.180	0.180	0.500	0.180	0.480	0.348
Ningxia	20	0.743	0.396	0.000	0.500	0.500	0.000	0.095	0.480	0.180	0.000	0.095	0.495	0.375	0.500	0.292
Xiapu	20	0.863	0.498	0.001	0.375	0.455	0.320	0.455	0.375	0.455	0.420	0.480	0.095	0.255	0.255	0.358
Yunnan	20	0.750	0.487	0.000	0.480	0.420	0.375	0.455	0.495	0.420	0.420	0.320	0.420	0.455	0.375	0.421
Pooled	140	0.748	0.457	0.000	0.474	0.446	0.293	0.446	0.420	0.493	0.311	0.426	0.408	0.500	0.389	

**Table 2:** Virulence frequencies and their contingency χ^2^ test in the seven *P. infestans* populations each composed of 20 genetically distinct isolates sampled from China.

Virulence factor	Fuzhou	Gansu	Guangxi	Guizhou	Ningxia	Xiapu	Yunnan	χ^2^test
*avr1*	0.15	0.60	0.05	0.55	0.50	0.25	0.60	26.89**
*avr2*	0.05	0.40	0.15	0.60	0.50	0.35	0.30	19.60**
*avr3a*	0.60	0.95	0.75	0.90	1.00	0.80	0.75	15.58*
*avr4*	0.40	0.75	0.40	0.85	0.95	0.65	0.65	23.64**
*avr5*	0.00	0.45	0.15	0.40	0.40	0.25	0.45	17.14**
*avr6*	0.40	0.60	0.25	0.70	0.90	0.35	0.70	26.11**
*avr7*	0.65	0.90	0.80	0.90	1.00	0.70	0.70	13.12*
*avr8*	0.30	0.85	0.45	0.90	0.95	0.60	0.80	34.50**
*avr9*	0.05	0.30	0.25	0.50	0.55	0.05	0.30	22.40**
*avr10*	0.20	0.70	0.25	0.90	0.75	0.15	0.65	44.72**
*avr11*	0.00	0.40	0.15	0.40	0.50	0.15	0.25	19.40**

**Table 3:** A comparison of population differentiation in virulence (*G*_*ST*_) and SSR marker (*F*_*ST*_) loci in the seven *Phytophthora infestans* populations sampled from China.

Virulence factor	G_ST_	T-test		Type of selection
		Value	P	
*avr1*	0.192	15.56	<0.0001	Diversifying
*avr2*	0.140	4.99	<0.0001	Diversifying
*avr3a*	0.111	−1.40	0.1646	Neutral
*avr4*	0.169	9.29	<0.0001	Diversifying
*avr5*	0.122	1.38	0.1707	Neutral
*avr6*	0.187	11.94	<0.0001	Diversifying
*avr7*	0.094	−5.72	<0.0001	Constraining
*avr8*	0.246	22.34	<0.0001	Diversifying
*avr9*	0.160	8.73	<0.0001	Diversifying
*avr10*	0.320	31.93	<0.0001	Diversifying
*avr11*	0.139	5.85	<0.0001	Diversifying

Positive t-values indicate that *G*_*ST*_ in virulence is larger than *F*_*ST*_ (=0.117) in SSR marker loci and vice versa.

**Table 4:** Meteorological data and race diversity measured with standardized Shannon index in the seven *Phytophthora infestans* populations each composed of 20 genetically distinct isolates sampled from China.

Population	AMT (C)^1^	Physiological race		
		Number	Shannon index	Complexity
Fuzhou	20.5	16	0.91	2.80 D
Gansu	11.7	19	0.98	6.90 AB
Guangxi	22.6	17	0.91	3.65 CD
Guizhou	14.7	17	0.97	7.60 AB
Ningxia	7.0	18	0.95	8.00 A
Xiapu	20.3	18	0.95	4.30 C
Yunnan	15.6	19	0.98	6.15 B
Pooled^2^	–	89	0.99	5.77

^1^Annual mean temperature

^2^When all isolates from different fields were combined.

**Table 5:** Correlation coefficients and their corresponding p values (in parentheses) between SSR heterozygosity and virulence diversity and between the frequency of virulence and the annual mean temperature (AMT) in the sites from which pathogens were collected.

Virulence factor	SSR heterozygosity		AMT
	Observed	Expected	
*avr1*	0.04 (0.932)	−0.09 (0.848)	−0.80 (0.031)
*avr2*	0.28 (0.543)	−0.04 (0.932)	−0.72 (0.068)
*avr3a*	0.13 (0.781)	0.45 (0.311)	−0.83 (0.021)
*avr4*	0.20 (0.667)	0.77 (0.043)	−0.89 (0.007)
*avr5*	0.12 (0.798)	−0.02 (0.966)	−0.75 (0.052)
*avr6*	0.12 (0.798)	0.70 (0.080)	−0.93 (0.002)
*avr7*	0.34 (0.456)	0.70 (0.080)	−0.79 (0.035)
*avr8*	0.40 (0.374)	0.63 (0.129)	−0.86 (0.013)
*avr9*	−0.53 (0.221)	−0.51 (0.242)	−0.78 (0.039)
*avr10*	−0.33 (0.470)	0.31 (0.499)	−0.81 (0.027)
*avr11*	−0.12 (0.798)	−0.28 (0.543)	−0.90 (0.006)
